# Admission rate for bronchiolitis of newborns and infants in Italian neonatal intensive care units in 2021: a survey of the Italian Society of Neonatology - Intensive Care of Early Childhood Study Group

**DOI:** 10.1186/s13052-025-01977-x

**Published:** 2025-06-17

**Authors:** Nunzia Decembrino, Roberta Leonardi, Tiziana Fedeli, Luana Conte, Chiara Distefano, Nicola Pozzi, Valeria Fichera, Ferdinando Spagnuolo, Camilla Gizzi, Fabio Mosca, Luigi Orfeo, Eloisa Gitto

**Affiliations:** 1 Neonatal Intensive Care Unit, University Hospital Policlinico “G. Rodolico San Marco”, University of Catania, Catania, Italy; 2 Postgraduate Training Programme in Pediatrics, Department of Clinical and Esperimental Medicine, University of Catania, Catania, Italy; 3 Neonatal Intensive Care Unit (T. F.), Fondazione Monza e Brianza per il Bambino e la sua Mamma and Azienda Socio-Sanitaria-Territoriale Monza, Monza, Italy; 4 Department of Physics and Chemistry, University of Palermo, Palermo, Italy; 5 Laboratory of Interdisciplinary Research Applied to Medicine (DReAM), University of Salento and ASL (Local Health Authority) Lecce (LE), Lecce, Italy; 6 Department of Maternal and Child Health, Neonatal Intensive Care Unit, San Pio Hospital, Benevento, Italy; 7 Department of Neonatology, University of Campania “Luigi Vanvitelli”, Napoli, Italy; 8 Department of Pediatrics and Neonatology, Ospedale Sandro Pertini Roma, Roma, Italy; 9 Fondazione IRCCS Ca’ Granda Ospedale Maggiore Policlinico, NICU, Milano, Italy; 10 Neonatology and Neonatal Intensive Care Unit, “San Giovanni Calibita” Fatebenefratelli Isola Tiberina Hospital Roma, Roma, Italy; 11 Department of Clinical and Experimental Medicine, Neonatal and Pediatric Intensive Care, University of Messina, Messina, Italy

**Keywords:** RSV bronchiolitis, Extended NICU, PICU shortage, Respiratory support

## Abstract

**Background:**

The shortage of Pediatric Intensive Care Unit (PICU) beds among some Italian regions is a major concern, especially during epidemics. During respiratory syncytial virus (RSV) bronchiolitis peak, Neonatal Intensive Care Units (NICU) often admit infants and toddlers requiring advanced respiratory support. We conducted a survey to quantify children hospitalized for RSV bronchiolitis in NICU in 2021 and to examine the adherence to treatment guidelines.

**Methods:**

Early Childhood Intensive Care Working Group of the Italian Society of Neonatology (SIN) conducted a survey distributed to SIN Network NICUs. The modified Delphi method was used to prepare the survey; duplicate responses were excluded. Analysis evaluated percentages.

**Results:**

Response rate was 67% (78/117 NICUs). Geographic distribution of responding centers was: 51% Southern-Islands, 38% North, 11% Center; 50% were Territorial Hospitals, 20% University Hospitals. Of respondents, 55% have 5–10 NICU beds; 70% routinely admit children > 44 weeks postconceptional age and > 28 days old, with a rate of < 10 toddlers/year in 50% of cases, 10–20 toddlers/year in 25% of cases and > 20 toddlers/year in 15%. In 2021, 40% of NICUs admitted < 10 bronchiolitis cases, 29% 11–20 cases. RSV was the leading cause of bronchiolitis. Reasons for NICU admission were respiratory distress syndrome (92%), feeding difficulties (58%), comorbidities (20%). High-flow oxygen (87%) and non-invasive ventilation (60%) were common respiratory supports provided; 10% of patients needed invasive ventilation. Treatment included inhaled steroids (46%), bronchodilators/systemic steroids (32%), antibiotics (40%); 60% of centers did not use sedation during NIV; 30% used midazolam, 13% dexmedetomidine, < 10% fentanyl.

**Conclusions:**

Our survey highlights that during the RSV epidemic, NICUs admitted toddlers to receive advanced respiratory support unavailable in pediatric ICUs. Most of the NICUs admitted fewer than 10 toddlers per year and less than 10 bronchiolitis, posing skill challenges for medical staff. This supports SIN’s proposal to identify some “extended NICUs” in regions with limited PICU beds, to centralize toddlers after an adequate training to gain knowledge/technical skills specific of pediatric critical care. This would help to overcome the PICU beds storage. Adherence to bronchiolitis management guidelines resulted suboptimal, with frequent but unrecommended use of inhaled steroids, bronchodilators, and antibiotics.

**Supplementary Information:**

The online version contains supplementary material available at 10.1186/s13052-025-01977-x.

## Introduction

The shortage of Pediatric Intensive Care Unit (PICU) beds is a pressing concern in Italy [1]. According to European standards, it is recommended to have one Pediatric Intensive Care Unit (PICU) bed for every 20,000–30,000 children. The ratio of PICU beds to the population of individuals aged 18 and younger in European countries varies widely, ranging from 0.5 to 11.7 beds per 100,000 children aged 1–18 years [2]. In Italy, the scenario is distant from the European recommended standards [3]. The shortage of beds in pediatric intensive care is particularly pronounced in the South, with a deficit of 67.3%, while in the North the deficit is 42.3% and in the Center 2.2% [1]. The consequence of this deficit is the inappropriate admission of numerous children to adult facilities, where they might not obtain the essential standards of care appropriate for their condition and age [4]. Critically ill children adequate for being treated in adult ICU should ideally be at least 12 years old and affected by conditions commonly found both in children and adults, such as community acquired sepsis or trauma [5]. Children with complex, pediatric-specific disorders are better served in PICUs. This is particularly true for toddlers, who represent 30% of PICU’s admission every year, because of reacutization of chronic conditions acquired during the perinatal period because of extreme prematurity, congenital disorders, perinatal hypoxic-ischemic syndromes [6].

As revealed by an unpublished survey carried out by the Italian Society of Neonatology (SIN) in 2015, particularly in regions of Italy lacking PICU beds, 66% of Neonatal Intensive Care Units (NICUs) had, over time, provided intensive care to infants and toddlers facing acute medical conditions, primarily respiratory (78% of cases), and to children with medical complexity. For this reason, the SIN proposed the institution of some “extended NICUs”, Neonatal Intensive Care Units specifically designated to provide intensive care not only to newborns but also to infants and toddlers with critical illnesses, particularly in general hospitals and in regions with a shortage of PICU beds. These units are intended to manage early childhood critical conditions sharing protocols with local PICUs, while cases requiring highly specialized care (such as neonatal or pediatric surgery, cardiac surgery, neurosurgery, severe burns, organ transplants) would be the competence of specialized pediatric hospitals [2]. Considering that it is mandatory for neonatologists working in an extended NICU to acquire specific technical and non-technical skills in pediatric intensive care medicine, SIN launched the Study Group on Early Childhood Intensive Care (TIPI) to promote the specific training in the management of critically ill children among all neonatologists interested to face with toddlers in their NICUs [6]. The shortage of PICUs beds is of significant concern particularly during epidemic periods [7], such as respiratory syncytial virus (RSV) bronchiolitis peak, putting pressure on PICU resources to accommodate infants requiring advanced respiratory support [8].

As the Early Childhood Intensive Care working party, we conducted a survey in 2021 to evaluate, in the real-world practice, the need to admit to the NICUs toddlers affected by RSV bronchiolitis, and the management of these cases in relation to national and international guidelines on bronchiolitis.

## Materials and methods

### Study design

In 2019, the TIPI Study Group of the SIN conducted a survey on “Extended Neonatal Intensive Care Units in Early Childhood” (unpublished data) to evaluate the rate of toddlers’ admission to Italian NICUs and the training needs among neonatologists involved. The survey was sent to 114 NICUs, with a response rate of 86% (98/114 NICUs).

The TIPI Study Group proposed a new survey in 2022, dedicated to the hospitalization rate for bronchiolitis in Italian NICUs in 2021, and the management of this condition. In February 2022, a web-based survey of 22 questions was developed to request feedback from the neonatal units. The survey was realized by the members of the TIPI Group (ND, EG, NP, TF) and evaluated by a panel of experts (LG, DT, CG, FS) in an online and anonymous reactive Delphi technique [9]. After three rounds in which the experts suggested changes to the proposed questions, consensus was reached on a final version of the survey. By the end of April 2022, the survey was finalized. On May 10, 2022, the first dispatch was made to 117 NICUs by the Survey Office of SIN. A reminder was sent to non-responders every 2 weeks for a maximum of three times; if no answer was received, participants were contacted by phone. The survey was closed in March 2023. The protocol was approved by the Institutional Review Board of Messina University (protocol number 0423).

### The questionnaire

The survey was composed of twenty-two questions: 12 multiple-choice questions with a single option, 5 questions allowing for multiple simultaneous responses, 5 open-ended questions. Questions focused on the rate of admission of infants and toddlers with RSV bronchiolitis in 2021, the treatment used, and the ventilatory strategy adopted; respondents were also asked to answer on the rate of admission of toddlers in their NICU every year, to compare this data with 2019 and the post-COVID pandemic. The complete list of the 22 questions is provided as Supplementary Material. The survey, which took approximately 10 min to complete, was sent to the chief of the NICUs participating in the SIN Network, with instructions for direct completion or completion by the expert in the field of each unit. A cover letter informed that participation in the survey was voluntary and anonymous. Information about the pertaining neonatal unit was asked to avoid duplicate responses. Responses were collected from neonatal units with only nurseries, to those NICUs extended to infants and those with pediatric intensive care beds (PICU).

### Statistical analysis

Data were collected through an electronic database (Microsoft 365 App for Enterprise^®^). Descriptive statistics were conducted on the entire dataset, presenting mean and standard deviation (SD) for continuous variables, and frequencies and percentages for categorical variables.

Respondents were further divided into three groups based on two criteria:

1. Geographic regions: N: “North,” C: “Center,” and S: “South and Islands”. North included Emilia-Romagna, Friuli-Venezia Giulia, Liguria, Lombardia, Piemonte, Trentino-Alto Adige, Valle d’Aosta, Veneto; Centre included Lazio, Marche, Toscana, Umbria; South and Islands included Abruzzo, Basilicata, Calabria, Campania, Molise, Puglia, Sardegna and Sicilia.
2. Number of Intensive Care Unit (ICU) beds: group A: < 5 ICU beds, group B: 6–10 ICU beds, group C: > 10 ICU beds.

To identify differences among these groups, the Kruskal-Wallis test was applied. Statistical significance was determined at a threshold of *p* < 0.05. All analyses were performed using SPSS software (version 29).

## Results

The survey was sent to 117 Neonatal Units from May 2022 to February 2023. Out of 117, 78 NICUs (67%) answered properly and have been enrolled in the final analysis. Demographic variables of the respondent NICUs and their geographical location are summarized in Tables 1 and 2 provides summary of NICU admission rate for bronchiolitis. Table 3 presents a comparison of responses among groups categorized by NICU size (small: <5 beds; medium: 6–10 beds; large: >10 beds). Similarly, Table 4 highlights differences in responses based on geographic location (North, Center, and South & Islands), illustrating variations in resource availability, patient management, and outcomes. Only statistically significant comparisons are reported. Full tables can be viewed in the supplementary materials.

**Table 1:** Demographic variables and geographical location corresponding to responding nicus

Demographic variables		*n* (%)
Geographic area*	North Center South	29 (37.1) 9 (11.5) 40 (51.3)
Type of Hospital	Territorial hospital University hospital IRCCS Accredited private hospital Others	36 (46.1) 16 (20.5) 5 (6.4) 8 (10.2) 13 (16.6)
Number of NICU beds	≤ 5 6–10 11–15 16–20 ≥ 20	13 (16.7) 43 (55.1) 16 (20.5) 5 (6.4) 1 (1.3)
Number of sub-intensive care beds	≤ 5 6–10 11–15 16–20 > 20 None	7 (9.2) 32 (42.1) 24 (31.6) 9 (11.8) 3 (3.9) 1 (1.3)
Isolation room availability	Yes No	71 (93.4%) 5 (6.6%)

*Italian Regions: North: Emilia-Romagna, Friuli-Venezia Giulia, Liguria, Lombardia, Piemonte, Trentino-Alto Adige, Valle d’Aosta, Veneto; Centre: Lazio, Marche, Toscana, Umbria; South: Abruzzo, Basilicata, Calabria, Campania, Molise, Puglia, Sardegna, Sicilia; IRCCS: Institute of Research and Cure of Scientific Interest

**Fig. 1:**
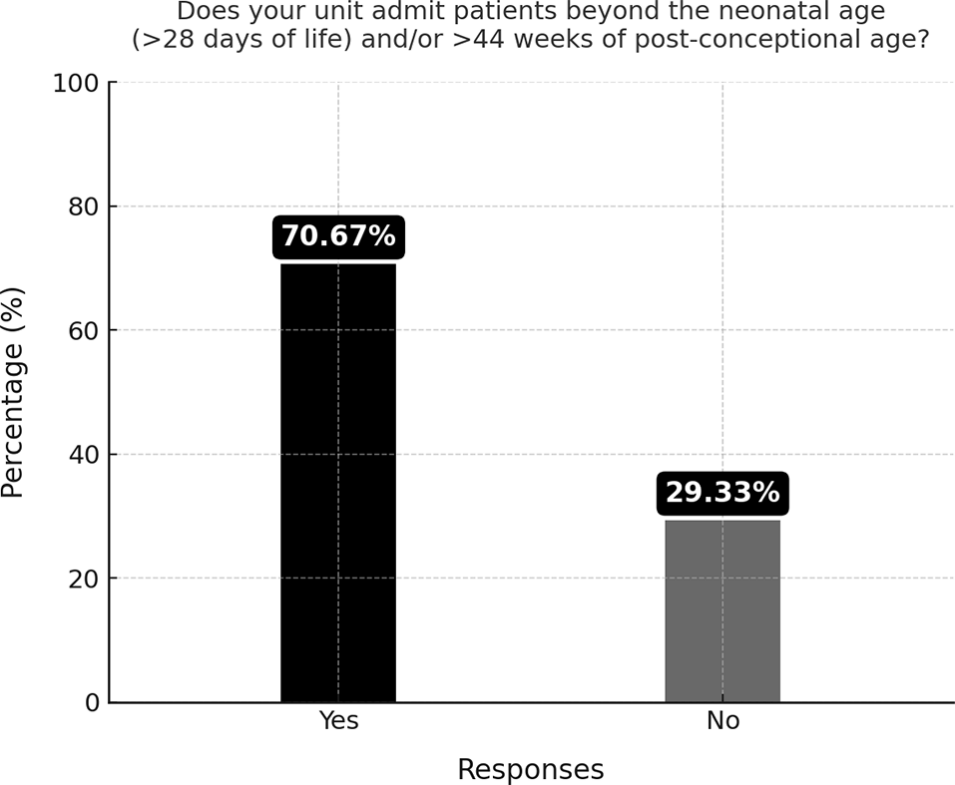
Percentage of units admitting patients beyond the neonatal age (> 28 days of life and/or > 44 weeks postconceptional age)

Admission of toddlers in neonatal units was found to be a common event, with about 71% of respondents (53 centers) declaring that patients over the neonatal age (> 28 days postnatal age and/or > 44 weeks postconceptional age) are admitted to their department (Fig. 1).

This data is in line with the survey conducted in 2019, when admission of toddlers in NICU was reported by 79% of respondents, in most cases (66%) for more than 10 years (unpublished data). In our survey, NICUs with more than 10 ICU beds admitted toddlers more frequently than small NICUs (less than 5 beds) (Table 3): 86,4% of NICUs with more than 10 ICU beds admitted toddlers compared to around 61% of NICUs with *≤* 10 ICU beds (Table 3). No differences were registered according to geographic areas (Table 4).

**Fig. 2:**
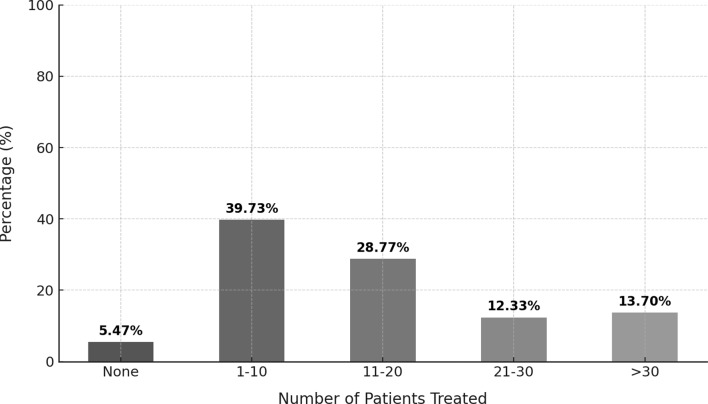
Percentage distribution of bronchiolitis cases treated in the units during 2021

When asked how many toddlers were admitted in NICU in 2021, overall 53% of centers reported fewer than 10 patients, 27% reported 11 to 20 patients, and 15% reported 21 to 50 patients (Table 2), with a difference among groups based on the number of ICU beds between NICUs with 6–10 ICU beds and NICUs with > 10 ICU beds and with a statistically significant difference between regions, with the majority of Northern NICUs admitting > 10 patients compared to Southern NICUs (Tables 3 and 4). When asked how many bronchiolitis were admitted in NICU in 2021, about 40% of centers reported a low rate of bronchiolitis admission, < 10 bronchiolitis/year (Table 2; Fig. 2).

**Table 2:** Summary of information reported by the nicus in relation to admission of toddlers and admission for bronchiolitis

	*n* (%)
Admission of patients over the age of newborn (> 28 days postnatal age and/or > 44 weeks postconceptional age	
1) Yes 2) No	53 (70.6) 22 (29.3)
Number of toddlers admitted in NICU in 2021	
1) ≤ 10 2) 11–20 3) 21–50 4) 51–100 5) > 100	39 (52,7) 20 (27) 11 (14.8) 2 (2.7) 2 (2.7)
Number of bronchiolitis admitted for bronchiolitis in NICU in 2021	
1) None 2) 1–10 3) 11–20 4) 21–30 5) > 30	4 (5.5) 29 (39.7) 21 (28.7) 9 (12.3) 10 (13.7)

Among the reported 958 patients with bronchiolitis admitted to Italian NICUs in 2021, 522 (54.5%) were newborns, 436 (45.5%) were infants > 44 weeks postmenstrual age or > 28 days postnatal age (PNA).

RSV was the leading cause of bronchiolitis, with only 8% of cases that tested negative to RSV. No differences in terms of viral agent were found according to geographic areas. The greatest incidence of bronchiolitis hospitalization occurred between October and December 2021 (81% of cases) without differences among regions (Table 4). Most of the patients admitted to NICUs were sent from the Emergency Pediatric Department and almost 60% of them were born in the same hospital. The main reason for NICU admission of patients with bronchiolitis was moderate-severe respiratory distress syndrome needing advanced respiratory support (91% of cases); the second most common reason (58%) was feeding difficulty accompanied by dehydration, followed by the onset of apnea (48%) and hypoxia (44%). Children admitted to NICU had comorbidities in 20% of cases, such as bronchopulmonary dysplasia, congenital heart disease, neuropathy, prematurity.

Through the questionnaire submitted we also aimed to investigate the therapeutic management of bronchiolitis in NICU. Most of the respondents (87%) used High Flow Nasal Oxygen (HFNO) as the first step of respiratory support, whilst only 23% of respondents used low flow oxygen. Switch to non-invasive ventilation (NIV) regarded 59% of patients, and invasive support 10% of them. No differences have been described on ventilatory support strategies among NICUs according to ICU beds, but NICUs in the North used more NIV and invasive support than NICUs in the South (Table 4). During NIV support, 60% of centers did not use any sedatives, while 30% administered midazolam, 13% dexmedetomidine and less than 10% used fentanyl for sedation. Our results showed that sedation was more common in NICUs in the North, where the most used drug during NIV appeared dexmedetomidine, while in the South around 90% of patients did not receive any sedation (Table 4). Almost 40% of respondents added antibiotic therapy to respiratory support, and more than 50% administered aerosolized saline hypertonic solution. Inhaled steroids and bronchodilators were used by 45% and 32% of respondents, respectively. No differences among ancillary therapies were reported according to ICU beds, nether to geographic distribution of NICUs. Mean duration of hospitalization of patients with bronchiolitis in NICU was 7.24 days (IQR 0–14 days; median 8 days), with no differences according to ICU beds nor geographic distribution of the center.

**Table 3:** Summary of the comparison of responses among groups based on the number of NICU beds (Group A: <5 beds; group B: 6–10 beds; group C: >10 beds), stratified by key variables. The frequency (N) and percentage of responses for each category are shown. The p-values represent overall comparisons and pairwise comparisons between groups (A vs. B, A vs. C, B vs. C). Statistical significance was set at *p* ≤ 0.05. Significant differences are indicated as follows: *p ≤ 0.05, **p ≤ 0.01, and ***p ≤ 0.001

Variable	Group A (*n* = 13, 16,6%)	Group B (*n* = 43, 55,1%)	Group C (*n* = 22, 28,2%)	*p* value			
				Overall	A vs. B	A vs. C	B vs. C
Does your unit admit patients beyond the neonatal age (> 28 days of life) and/or > 44 weeks of postconceptional age?				NS	NS	0.04*	0.02*
Yes No missing	8 (61.5) 5 (38.5) 0	26 (60.5) 15 (34.9) 2 (4.7)	19 (86.4) 2 (9.1) 1 (4.5)				
How many patients beyond the neonatal age were admitted in 2021?				NS	NS	NS	0.03*
< 10 10–20 20–50 50–100 > 100 missing	7 (53.8) 3 (23.1) 3 (23.1) 0 0 0	26 (60.5) 10 (23.3) 4 (9.3) 1 (2.3) 0 2 (4.7)	6 (27.3) 7 (31.8) 4 (18.2) 1 (4.5) 2 (9.1) 2 (9.1)				
How many children with bronchiolitis were older than 28 days of life or 44 weeks postconceptional age at the time of admission?				0.04*	NS	NS	0.02*
None < 5 5–10 > 10 missing	0 6 (46.1) 1 (7.7) 5 (38.4) 1 (7.7)	12 (27.9) 11 (25.5) 9 (20.9) 7 (16.2) 4 (9.3)	0 6 (27.2) 6 (27.2) 7 (31.8) 3 (13.6)				

**Table 4:** Summary of the comparison of responses among groups based on geographic location (Group N: North; group C: center; group S: South & Islands), stratified by key variables. The table presents the frequency (N) and percentage of responses for each category across geographic regions. The p-values represent overall comparisons and pairwise comparisons between groups (N vs. C, N vs. S, and C vs. S). Statistical significance was set at *p* ≤ 0.05. Significant differences are indicated as follows: *p ≤ 0.05, **p ≤ 0.01, and ***p ≤ 0.001

Variable	N (*n* = 29, 37.1%)	C (*n* = 9, 11.5%)	S (*n* = 40, 51.3%)	*p* value			
				Overall	*N* vs. C	*N* vs. S	C vs. S
**Does your unit admit patients beyond the neonatal age (> 28 days of life) and/or > 44 weeks of post-conceptional age?**				NS	0.04*	NS	NS
Yes No missing	23 (79.3) 4 (13.8) 2 (6.9)	4 (44.4) 4 (44.4) 1 (11.1)	26 (65.0) 14 (35.0) 0				
**How many patients beyond the neonatal age were admitted in 2021?**				0.005**	NS	0.001***	NS
< 10 10–20 20–50 50–100 > 100 missing	7 (24.1) 12 (41.4) 5 (17.2) 2 (6.9) 0 3 (10.3)	6 (66.7) 0 2 (22.2) 0 0 1 (11.1)	26 (65.0) 8 (20.0) 4 (10.0) 0 2 (5.0) 0				
**What type of ventilatory support did you predominantly use to treat RSV + bronchiolitis patients?**				0.05*	NS	0.01**	NS
Low-flow oxygen therapy High-flow oxygen therapy with nasal cannulas (HFNC) Non-invasive ventilation Invasive ventilation	2 (4.2) 22 (46.8) 18 (38.3) 5 (10.6)	5 (33.3) 6 (40.0) 4 (26.6) 0	9 (14.5) 32 (51.6) 19 (30.6) 2 (3.2)				
**Did you use sedation for RSV + bronchiolitis patients on non-invasive ventilatory support? If yes**,** which one?**				0.05*	NS	0.03*	NS
None Midazolam Dexmedetomidine missing	17 (58.6) 1 (3.4) 4 (13.8) 7 (24.1)	7 (77.8) 0 0 2 (22.2)	37 (92.5) 2 (5.0) 0 1 (2.5)				

## Discussion

Bronchiolitis is one of the main viral infections of the lower respiratory tract in children [10]. About 3% of cases require hospitalization. RSV infection represents the leading cause of bronchiolitis. Main risk factors for hospitalization due to RSV infection are age < 1 year and birth during epidemic season [11]. Of children who require hospitalization, 18% is under one year of age; 3 to 6% of these children require admission to the ICU for advanced respiratory support, representing about 13% of annual PICU admissions [12]. Based on age and local organization, across Europe patients with severe bronchiolitis are admitted to neonatal, pediatric, or mixed ICUs taking care of both adults and pediatric patients. Our study reveals critical insights into the RSV epidemic’s impact on pediatric healthcare in Italy, highlighting the strain on NICUs due to high admission rates of toddlers with bronchiolitis. This survey was sent to all the Italian NICUs members of the Italian Society of Neonatology, and response was voluntary. The response rate was relatively high, covering almost 67% of the NICUs. However, the geographical distribution of the NICUs that responded was unbalanced, with a prevalence of the South/Islands. Nevertheless, this should not limit the generalizability of the results to the whole Italian territory considering that admission rate of toddlers among NICUs did not present geographical differences. Indeed, Northern NICUs, where most of the PICUs are located, also admitted toddlers with bronchiolitis. This confirms that PICU beds in Italy are insufficient to face the increased needs during epidemics, similarly to what happened during COVID-19 pandemics for adult ICU beds. These results further highlight the importance of programming strategies aimed to reduce the average length of stay in PICU and NICU, favoring transfer to departments with lower intensity of care, as recently proposed by De Luca et al. [8]. Of particular importance, our survey highlights that the hospitalization of infants and toddlers occurs in a dispersed and random manner in the Italian NICUs, based on bed’s availability rather than on the ability of the staff to take care of a patient who is no longer a newborn and therefore requires different knowledges and skills of pediatric intensive care (e.g. in the central line management or in the monitoring of the critically ill child) as well as dedicated facilities (e.g. dedicated beds, ventilators appropriate for the child’s weight and appropriate NIV interfaces). Indeed, most respondents declared that they regularly admit toddlers, but less than 10 per year; the bronchiolitis admission rate follows the same trend, with centers belonging to the same region or area that admitted fewer than 5 children with bronchiolitis in the year. As discussed in the recent work of De Luca [8], the lack of clinical guidelines dedicated to the management of bronchiolitis in ICU poses problems related to the difference of clinical backgrounds and levels of preparedness to work with older patients and to prevent nosocomial cross-contamination during outbreaks, resulting in different ICU management, with potential worse outcomes, prolonged length of stay in ICU and increased costs. A useful strategy to address these critical issues could be to support the areas with the greatest shortage of PICU beds with some “extended NICUs” with adequately trained clinical staff, where critical toddlers can be centralized. The extended NICU model could offer several advantages. It would help to reduce intraregional and interregional transport of critical infants, that entails additional costs for the health system and for families, in terms of travel, accommodation, lost working days, not to mention the intangible costs of being far from loved ones and support networks. Furthermore, the pediatric transport service with dedicated personnel is not active in all regions of Italy and neonatal transport, in a non-homogeneous manner between regions, only covers the age group within one month of life or, more rarely, up to 6 kg of weight. This means that the transport of the infant is often delegated to the adult emergency transport service, which has no expertise in the cure of the smallest child. Centralization of patients in dedicated extended NICUs is also detrimental to reduce costs related to dedicated facilities and consumable materials related to toddlers and, most important, it would facilitate the staff maintenance of the necessary manual and theoretical skills, thanks to an adequate number of infants admitted per year. Literature data, indeed, have shown that critically ill children have better outcomes and lower mortality if admitted to PICU compared to adult ICUs and that high-volume patients ICUs provide better care than low-volume ones [4, 13]. There is an increasing number of children with medical complexity, resulting from improved life expectancy of children with chronic diseases due to prematurity, perinatal asphyxia, congenital disorders (e.g. cerebral palsy, bronchopulmonary dysplasia, metabolic disorders), who represent the greater part of infants who need PICU care. Neonatologists are more familiar with the management of these diseases than adult intensivists. NICUs are widely and equally distributed throughout Italy, making it possible to identify some of these to specialize as extended NICU. Managing toddlers in extended NICUs would help to attain the target of occupancy of 85% of PICU beds, freeing up PICU’s resources for older patients, while ensuring that younger children would be cared for in pediatric environments with nurses skilled in the management of infants and family needs. Specialized staff can reduce the length of hospital stay thanks to adequate care, further reducing costs. In the case of new pandemics, or during epidemics like RSV ones, this could be of particular importance.

Recent studies have highlighted an increased burden of hospitalizations for bronchiolitis in the post-COVID pandemic era, with a rise in ICU admissions and associated healthcare costs [14–16]. The introduction of non-pharmaceutical interventions (NPIs) during the COVID-19 pandemic has also markedly influenced the typical seasonality and incidence rates of RSV bronchiolitis, leading to a notable 45% decrease in hospital admissions and a shorter duration of hospital stays during COVID-19 spread and a surge of viral infections in the immediate post-COVID season because of an immunity debt [17, 18]. In France, RSV peak in 2020–2021 was delayed in the period usually corresponding to the end of the epidemic [19]. In Australia, during the COVID-19 pandemic RSV activity was absent, with a marked and unusual resurgence of RSV activity 6 months later, during the summer season [20]. In Lombardy, northern Italy, during the pandemic winter season 2020–2021 no cases of RSV were detected [21]. In our cohort, because of the discontinuation of COVID-isolation measures imposed in 2019–2020 fall (physical distancing, the use of face masks, and the discontinuation of in-person teaching activities), RSV bronchiolitis peaked between October 2021 and December 2022, differently from Italian epidemiologic data reporting usual RSV peak between January and February. This atypical pattern, that reflects changes in herd immunity, viral interference, or altered immunological response due to a presumed immunity theft, underscores the need for healthcare systems to identify strategies to cope with variable demands and to adequately organize facilities and staff to manage outbreaks, including providing adequate training plans that concern infection control strategies to avoid viral spread (isolation rooms, ideally with negative pressure ventilation, staff cohorting, use of individual protective disposables, and hand hygiene practices extended to parents).

Our survey also aimed to analyze bronchiolitis treatment strategies adopted in Italian NICUs. ICU admission was related to advanced respiratory support needs. The use of high flow nasal oxygen therapy was not sufficient in most cases, with other ventilatory strategies, such as CPAP and NIV, used in almost 60% of reported cases. This is in line with recent literature data [22, 23] and further stress the need for adequate medical training considering that bronchiolitis pattern is often mixed, with restrictive/mixed and obstructive lung areas, deserving solid expertise in ventilatory strategies [23]. A recent Finnish population-based study by Selin et al. evaluating the management of bronchiolitis in PICUs before and after the publication of national bronchiolitis guidelines in 2015 reported a high prevalence of HFNC usage. Specifically, 94% of patients received high-flow oxygen therapy (HFOT), 37% were treated with nasal continuous positive airway pressure (CPAP), and only 9% required mechanical ventilation [24]. Our findings suggest a lower utilization of HFNC in Italian NICUs, which may have contributed to the progression to more severe pulmonary conditions that required more intensive support, such as CPAP and NIV. The lower usage of HFNC observed in our study might indicate a gap in training or resource allocation, underscoring the importance of improving the availability and proper use of non-invasive respiratory strategies to optimize outcomes for patients with bronchiolitis. Dehydration due to failure to thrive was the second most frequent cause of ICU admission. Neonatologists are prone to use epicutaneo-cava catheters in newborns, which are not adequate for older and critically ill patients. Specific training for the choice of the most appropriate venous access and for central venous catheter (CVC) placement is warranted [25]. The use of Point of Care Ultrasound should also be implemented in NICUs to help in differential diagnosis between bronchiolitis, acute respiratory distress syndrome, and pneumonia [26–29], in evaluating cardiac filling (preload assessment) and intravascular volume status, and to help in central-line placement [30, 31]. Our survey did not question on the difficulties perceived by professionals in managing infants and toddlers in NICU, nether analyzed the need for staff training because it is a topic that had been covered in a previous survey (unpublished data) aimed at focusing on training needs and planning the TIPI study group’s training plan.

Our data also highlight a wide use of beta-2 agonists and antibiotics. These ancillary treatments lack strong evidence and are not warranted by Italian and international guidelines focusing on bronchiolitis management in pediatric wards or emergency departments [32–36]. Low adherence to bronchiolitis treatment guidelines both in the outpatients and hospital setting in Italy has already been reported [37–40], but it is the first time that NICUs are involved in such analyses. Against the use of inhaled beta-2 agonists among patients affected by bronchiolitis there is evidence of their ineffectiveness in modifying the outcome, since wheezing depends above all on the obstruction of the bronchioles by mucus, rather than on bronchospasm. Beta2-agonists, while commonly administered in emergency departments, do not reduce admission rates for children with bronchiolitis [41]. Furthermore, beta2-agonists, particularly when administered intravenously, have been associated to adverse events like tremor, hypokalaemia, and supraventricular tachycardia [42]. However, several phenotypes of bronchiolitis exist, depending on viral agent involved, family history of atopy, and patient’s age > 6 months [43, 44], so it is reasonable to attempt a trial in more critical patients, especially those with predominant wheezing and dyspnea. Our study was not powered to differentiate strategy treatment between infants and toddlers, but it is plausible that patients requiring ICU experience a worse alveolar damage and that beta-2 agonists were used in older patients. The widespread use of antibiotics in NICU should be discouraged to reduce the burden of antibiotic resistance since they do not improve the outcome in non-invasively ventilated patients with bronchiolitis [45], but bacterial co-infection could be a leading cause of ICU admission. The burden of multidrug-resistant organisms (MDROs) in NICUs is particularly alarming, with infections caused by resistant pathogens accounting for significant morbidity and mortality, and an estimated annual economic impact in Europe exceeding 1.1 billion Euro, causing over 33,000 deaths annually [46, 47]. However, we have no data to understand if antibiotic-therapies used by respondents in our survey were a continuation of a course already prescribed by the general pediatrician or if pneumonia or another bacterial infection was the reason to start antibiotic in NICU. Further studies should analyze this issue.

To our knowledge, this is the first Italian study focusing on the rate of admission to NICU of newborns and toddlers affected by bronchiolitis. The main limitations of our study include the survey response rate and potential biases due to open-ended questions, since many did not respond by reporting a numerical data, as requested, but with comments, or with percentages or ranges of values, rendering difficult to categorize the answers on the type of ventilatory support used.

Certainly, our study paves the way for several research trajectories. Understanding and addressing the implications of RSV bronchiolitis on PICU resources is of paramount importance to mitigate the strain on the healthcare system, considering the growing demand for advanced respiratory support [48–50]. In children below the age of 1 year, RSV represents the first cause of death among respiratory infections and the first cause of hospitalization [51], so enhanced surveillance, emphasis on infection prevention measures, and targeted interventions for high-risk populations are essential. In the next future, extended prophylaxis with monoclonal antibodies against RSV could help to reduce the burden of RSV epidemics [52], but bronchiolitis per se may be sustained by different viral agents and the recent COVID-pandemic has taught us the need to have a resilient healthcare system, ready to handle the sudden increase in ICU needs. The Extended NICU model addresses the dual challenges of PICU bed shortages and the need for adept management of older infants and toddlers. This model may improve long-term treatment efficacy by ensuring that infants and toddlers receive care in environments with specialized staff adequately trained, ameliorating clinical outcome for young patients requiring advanced respiratory support. In addition, by centralizing toddlers’ care in extended NICUs, healthcare systems may achieve costs saving through reduced patient transfer and optimized resources use, while allowing staff to maintain expertise through regular management of critically ill infants and toddlers.

## Conclusions

This survey provides insight into admission rate of newborns and toddlers to NICU during bronchiolitis epidemics in a large sample of Italian hospitals. We propose the “Extended-NICU model” to deal with the problem of PICU resources allocation during epidemics. Longitudinal studies are needed to monitor RSV epidemiology and admission rate to PICU and NICU of children with bronchiolitis, to compare the outcomes of toddlers admitted to NICU versus PICU and to analyze the efficacy of treatment protocols used. This study also emphasizes the importance of bronchiolitis treatment guideline adherence and the need of adopting national or European guidelines to standardize the care of children with bronchiolitis admitted to ICUs.

## Electronic supplementary material

Below is the link to the electronic supplementary material.

Supplementary Material 1

## Data Availability

The datasets generated and analyzed during the current study are available from the corresponding author on reasonable requests.

## Abbreviations

RSV: Respiratory Syncytial Virus
NICU: Neonatal Intensive Care Unit
PICU: Pediatric Intensive Care Unit
NIV: Non-Invasive Ventilation
HFNO: High Flow Nasal Oxygen
CVC: Central Venous Catheter
IRCCS: Institute of Research and Cure of Scientific Interest
SIN: Italian Society of Neonatology
TIPI: Intensive Care of Early Childhood Study Group
NPIs: Non-Pharmaceutical Interventions
