# Supplementary material for: Admission rate for bronchiolitis of newborns and infants in Italian neonatal intensive care units in 2021: a survey of the Italian Society of Neonatology - Intensive Care of Early Childhood Study Group
Source: Ital J Pediatr. 2025 Jun 17;51:192. doi: 10.1186/s13052-025-01977-x (PMC12175376; doi:10.1186/s13052-025-01977-x)
Supplement: Supplementary file 1 — Supplementary Material 1 [file 13052_2025_1977_MOESM1_ESM.docx]

**Supplementary Material**

1. **Survey Questions**
2. **Specify the name of the hospital to which the respondent belongs (**Open-ended response**)**
3. **Indicate the city where the hospital is located. (**Open-ended response**)**
4. **Geographical area where the hospital is located. Select one of the following options:**
   - North (Valle d'Aosta, Piemonte, Lombardia, Liguria, Veneto, Alto Adige, Friuli-Venezia Giulia, Emilia-Romagna)
   - Center (Toscana, Marche, Umbria, Lazio)
   - South and Islands (Abruzzo, Molise, Puglia, Basilicata, Calabria, Campania, Sardegna, Sicilia)
5. **Institution of affiliation. Select one of the following options:**
   - Territorial Hospital (ASL, AUSL, ASP, ASM, ATS)
   - University Hospital
   - IRCCS (Institute for Research and Cure of Scientific Interest)
   - Accredited Private Hospital
   - Other (please specify)
6. **How many Intensive Care Unit (ICU) beds are available in your unit?**
   - <5
   - 5-10
   - 10-15
   - 15-20
   - > 20
7. **How many Sub-Intensive Care beds are available in your unit?**
   - <5
   - 5-10
   - 10-15
   - 15-20
   - > 20
   - None
8. **Are isolation beds available in your unit?**
   - Yes
   - No
9. **Does your unit admit patients beyond the neonatal age (>28 days of life) and/or >44 weeks of postconceptional age?**
   - Yes
   - No
10. **How many patients beyond the neonatal age were admitted in 2021?**
    - <10
    - 10-20
    - 20-50
    - 50-100
    - > 100
11. **How many patients with bronchiolitis were treated in your unit in 2021?**
    - None
    - 1-10
    - 11-20
    - 21-30
    - > 30
12. **How many children with bronchiolitis were younger than 28 days of life at the time of admission? Provide the number of children. (**Open-ended response**)**
13. **How many children with bronchiolitis were older than 28 days of life or 44 weeks of postconceptional age at the time of admission? Provide the number of children. (**Open-ended response**)**
14. **In how many of these children with bronchiolitis was RSV isolated?**
    - None
    - 1-10
    - 11-20
    - 21-30
    - > 30
15. **What was the period of highest incidence of hospitalizations for RSV-positive bronchiolitis?**
    - October-December
    - January-February
    - March-May
16. **To which of the following categories do the RSV+ patients admitted to your unit belong?** (multiple answers possible)
    - Born in your hospital (including ex-premature infants)
    - Born in other hospitals and later transferred (including ex-premature infants)
    - Admitted from the Emergency Department/Pediatrics of your hospital
    - Transferred from the Emergency Department/Pediatrics of another hospital
    - Other (please specify)
17. **What was the reason for ICU admission of children with RSV+ bronchiolitis? (multiple answers possible):**
    - Hypoxia
    - Moderate-to-severe respiratory distress requiring ventilatory support (CPAP or invasive ventilation)
    - Apnea episodes
    - Feeding difficulties and dehydration
    - Risk factors (e.g., bronchopulmonary dysplasia, congenital heart disease, neuropathy, prematurity)
18. **What type of ventilatory support did you predominantly use to treat RSV+ bronchiolitis patients?** (multiple answers possible)
    - Low-flow oxygen therapy
    - High-flow oxygen therapy with nasal cannulas (HFNC)
    - Non-invasive ventilation
    - Invasive ventilation
19. **How many children were treated with non-invasive ventilatory support other than High Flow Nasal Cannula (HFNC)? Provide the number of children. (**Open-ended response**)**
20. **How many children were treated with invasive ventilatory support?Provide the number of children. (**Open-ended response**)**
21. **What pharmacological therapies did you use to treat RSV+ bronchiolitis patients? (multiple answers possible):**
    - No pharmacological therapy, only ventilatory support
    - Hypertonic saline solution
    - Systemic steroids
    - Inhaled steroids
    - Inhaled bronchodilators
    - Endotracheal surfactant
    - Antibiotic therapy
    - Inotropes
    - Other (please specify)
22. **Did you use sedation for RSV+ bronchiolitis patients on non-invasive ventilatory support? If yes, which one?** (multiple answers possible)
    - None
    - Midazolam
    - Dexmetedomidina
    - Other (please specify)
23. **What was the average length of stay (in days) in the NICU for children with RSV+ bronchiolitis? Provide the average number of days. (**Open-ended response**)**

**B.**

**Table 3. Comparison of responses among groups based on the number of NICU beds (Group A: <5 beds; Group B: 6–10 beds; Group C: >10 beds), stratified by key variables.
The frequency (N) and percentage of responses for each category are shown. The p-values represent overall comparisons and pairwise comparisons between groups (A vs. B, A vs. C, B vs. C). Statistical significance was set at p ≤ 0.05. Significant differences are indicated as follows: *p ≤ 0.05, **p ≤ 0.01, and ***p ≤ 0.001. NS indicates non-significant results.**

| **Variable** | **Group A**  **(n = 13, 16,6%)** | **Group B**  **(n = 43, 55,1%)** | **Group C**  **(n = 22, 28,2%)** | ***p value*** | | | |
| --- | --- | --- | --- | --- | --- | --- | --- |
|  |  |  |  | **Overall** | **A vs B** | **A vs C** | **B vs C** |
| **How many Sub-Intensive Care beds are available in your unit?**  None  <5  5-10  >10  missing | 0  3 (23.1)  8 (61.5)  2 (15.4)  0 | 1 (2.3)  4 (9.3)  16 (37.2)  21 (48.8)  1 (2.3) | 0  0  13 (59.1)  8 (36.4)  1 (4.5) | NS | **0.05*** | **0.03*** | NS |
| **Are isolation beds available in your unit?**  Yes  No  missing | 12 (92.3)  1 (7.7)  0 | 39 (90.7)  3 (7.0)  1 (2.3) | 20 (90.9)  1 (4.5)  1 (4.5) | NS | NS | NS | NS |
| **Does your unit admit patients beyond the neonatal age (>28 days of life) and/or >44 weeks of postconceptional age?**  Yes  No  missing | 8 (61.5)  5 (38.5)  0 | 26 (60.5)  15 (34.9)  2 (4.7) | 19 (86.4)  2 (9.1)  1 (4.5) | NS | NS | **0.04*** | **0.02*** |
| **How many patients beyond the neonatal age were admitted in 2021?**  <10  10-20  20-50  50-100  >100  missing | 7 (53.8)  3 (23.1)  3 (23.1)  0  0  0 | 26 (60.5)  10 (23.3)  4 (9.3)  1 (2.3)  0  2 (4.7) | 6 (27.3)  7 (31.8)  4 (18.2)  1 (4.5)  2 (9.1)  2 (9.1) | NS | NS | NS | **0.03*** |
| **How many patients with bronchiolitis were treated in your unit in 2021?**  None  1-10  11-20  21-30  >30  missing | 0  5 (38.5)  5 (38.5)  0  2 (15.4)  1 (7.7) | 4 (9.3)  14 (32.6)  11 (25.6)  7 (16.3)  5 (11.6)  2 (4.7) | 0  10 (45.5)  5 (22.7)  2 (9.1)  3 (13.6)  2 (9.1) | NS | NS | NS | NS |
| **How many children with bronchiolitis were younger than 28 days of life at the time of admission? (open answer)**  None  <5  5-10  >10  missing | 1 (7.7)  6 (46.1)  2 (15.4)  3 (23.1)  1 (7.7) | 6 (13.9)  9 (20.1)  8 (18.6)  18 (41.8)  2 (4.7) | 0  8 (36.4)  2 (9.0)  8 (36.4)  4 (18.1) | NS | NS | NS | NS |
| **How many children with bronchiolitis were older than 28 days of life or 44 weeks postconceptional age at the time of admission? (open answer)**  None  <5  5-10  >10  missing | 0  6 (46.1)  1 (7.7)  5 (38.4)  1 (7.7) | 12 (27.9)  11 (25.5)  9 (20.9)  7 (16.2)  4 (9.3) | 0  6 (27.2)  6 (27.2)  7 (31.8)  3 (13.6) | **0.04*** | NS | NS | **0.02*** |
| **In how many of these children with bronchiolitis was RSV isolated?**  None  1-10  11-20  21-30  >30  missing | 2 (15.4)  4 (30.8)  6 (46.2)  0  0  1 (7.7) | 5 (11.6)  17 (39.5)  12 (27.9)  3 (7.0)  2 (4.7)  4 (9.3) | 1 (4.5)  10 (45.5)  3 (13.6)  3 (13.6)  3 (13.6)  2 (9.1) | NS | NS | NS | NS |
| **What was the period of highest incidence of hospitalizations for RSV-positive bronchiolitis?**  January-February  March-May  October-December  Missing | 1 (7.7)  1 (7.7)  10 (76.9)  1 (7.7) | 5 (11.6)  0  33 (76.7)  5 (11.6) | 5 (22.7)  1 (4.5)  14 (63.6)  2 (9.1) | NS | NS | NS | **0.05*** |
| **To which of the following categories do the RSV+ patients admitted to your unit belong?**  Born in your hospital (including ex-premature infants)  Born in other hospitals and later transferred (including ex-premature infants)  Admitted from the Emergency Department/Pediatrics of your hospital  Transferred from the Emergency Department/Pediatrics of another hospital | 9 (31.0)  5 (17.2)  8 (27.6)  7 (24.1) | 19 (22.0)  20 (23.2)  26 (30.2)  21 (24.4) | 13 (28.2)  10 (21.7)  15 (32.6)  8 (17.4) | NS | NS | NS | NS |
| **What was the reason for ICU admission of children with RSV+ bronchiolitis?**  Hypoxia  Moderate-to-severe respiratory distress requiring ventilatory support (CPAP or invasive ventilation)  Apnea episodes  Feeding difficulties and dehydration  Risk factors (e.g., bronchopulmonary dysplasia, congenital heart disease, neuropathy, prematurity) | 6 (22.2)  9 (33.3)  3 (11.1)  7 (25.9)  2 (7.4) | 17 (16.2)  35 (33.3)  22 (20.9)  22 (20.9)  9 (8.6) | 8 (16.3)  19 (39.8)  8 (16.3)  11 (22.4)  3 (6.1) | NS | NS | NS | NS |
| **What type of ventilatory support did you predominantly use to treat RSV+ bronchiolitis patients?**  Low-flow oxygen therapy  High-flow oxygen therapy with nasal cannulas (HFNC)  Non-invasive ventilation  Invasive ventilation | 5 (25.0)  10 (50.0)  4 (20.0)  1 (5.0) | 10 (13.7)  32 (43.8)  25 (34.2)  6 (8.2) | 1 (3.2)  18 (58.0)  12 (38.7)  0 | NS | NS | NS | NS |
| **How many children were treated with non-invasive ventilatory support other than High Flow Nasal Cannula (HFNC)? (open answer)**  None  <5  5-10  >10  missing | 5 (38.4)  3 (23.0)  4 (30.7)  0  1 (7.7) | 5 (11.6)  14 (32.5)  5 (11.6)  11 (25.6)  8 (18.6) | 1 (4.5)  8 (36.4)  7 (31.8)  3 (13.6)  3 (13.6) | NS | NS | NS | NS |
| **How many children were treated with invasive ventilatory support? (open answer)**  None  <5  5-10  >10  missing | 10 (76.9)  2 (15.4)  0  0  1 (7.7) | 18 (41.9)  16 (37.2)  1 (2.3)  1 (2.3)  7 (16.2) | 11 (50.0)  8 (36.4)  0  0  3 (13.6) | NS | NS | NS | NS |
| **What pharmacological therapies did you use to treat RSV+ bronchiolitis patients?**  No pharmacological therapy, only ventilatory support  Hypertonic saline solution  Systemic steroids  Inhaled steroids  Inhaled bronchodilators  Endotracheal surfactant  **Antibiotic therapy**  **Inotropes** | 3 (8.1)  8 (21.6)  5 (13.5)  8 (21.6)  8 (21.6)  1 (2.7)  4 (10.8)  0 | 17 (19.3)  20 (22.7)  11 (12.5)  13 (14.8)  9 (10.2)  2 (2.2)  14 (15.9)  2 (2.2) | 6 (13.6)  9 (20.4)  5 (11.4)  10 (22.7)  5 (11.4)  0  9 (20.4)  0 | NS | NS | NS | NS |
| **Did you use sedation for RSV+ bronchiolitis patients on non-invasive ventilatory support? If yes, which one?**  None  Midazolam  Dexmedetomidina | 10 (83.3)  2 (16.7)  0 | 22 (57.9)  11 (28.9)  5 (13.1) | 10 (47.6)  7 (33.3)  4 (19.2) | NS | NS | NS | NS |
| **What was the average length of stay (in days) in the NICU for children with RSV+ bronchiolitis? (open answer)**  None  <5  5-10  >10  Missing | 0  2 (15.4)  9 (69.2)  1 (7.7)  1 (7.7) | 2 (4.7)  2 (4.7)  5 (11.6)  11 (25.6)  8 (18.6) | 0  8 (18.6)  30 (69.8)  1 (2.3)  4 (9.3) | NS | NS | NS | NS |

**C.**

**Table 4. Comparison of responses among groups based on geographic location (Group N: North; Group C: Center; Group S: South & Islands), stratified by key variables.
The table presents the frequency (N) and percentage of responses for each category across geographic regions. The p-values represent overall comparisons and pairwise comparisons between groups (N vs. C, N vs. S, and C vs. S). Statistical significance was set at p ≤ 0.05. Significant differences are indicated as follows: *p ≤ 0.05, **p ≤ 0.01, and ***p ≤ 0.001. NS indicates non-significant results.**

| **Variable** | **N**  **(n = 29, 37.1%)** | **C**  **(n = 9, 11.5%)** | **S**  **(n = 40, 51.3%)** | ***p value*** | | | |
| --- | --- | --- | --- | --- | --- | --- | --- |
|  |  |  |  | **Overall** | **N vs C** | **N vs S** | **C vs S** |
| **How many Sub-Intensive Care beds are available in your unit?**  None  <5  5-10  >10  missing | 1 (3.4)  4 (13.8)  7 (24.1)  16 (55.1)  1 (3.4) | 0  0  5 (55.6)  3 (33.3)  1 (11.1) | 0  3 (7.5)  20 (50.0)  17 (42.5)  0 | NS | NS | NS | NS |
| **Are isolation beds available in your unit?**  Yes  No  missing | 27 (93.1)  1 (3.4)  1 (3.4) | 8 (88.9)  0  1 (11.1) | 36 (90.0)  4 (10.0)  0 | NS | NS | NS | NS |
| **Does your unit admit patients beyond the neonatal age (>28 days of life) and/or >44 weeks of post-conceptional age?**  Yes  No  missing | 23 (79.3)  4 (13.8)  2 (6.9) | 4 (44.4)  4 (44.4)  1 (11.1) | 26 (65.0)  14 (35.0)  0 | NS | **0.04*** | NS | NS |
| **How many patients beyond the neonatal age were admitted in 2021?**  <10  10-20  20-50  50-100  >100  missing | 7 (24.1)  12 (41.4)  5 (17.2)  2 (6.9)  0  3 (10.3) | 6 (66.7)  0  2 (22.2)  0  0  1 (11.1) | 26 (65.0)  8 (20.0)  4 (10.0)  0  2 (5.0)  0 | **0.005**** | NS | **0.001***** | NS |
| **How many patients with bronchiolitis were treated in your unit in 2021?**  None  1-10  11-20  21-30  >30  missing | 2 (6.9)  12 (41.4)  6 (20.7)  3 (10.3)  2 (6.9)  4 (13.8) | 1 (11.1)  3 (33.3)  2 (22.2)  0  2 (22.2)  1 (11.1) | 1 (2.5)  14 (35.0)  13 (32.5)  6 (15.0)  6 (15.0)  0 | NS | NS | NS | NS |
| **How many children with bronchiolitis were younger than 28 days of life at the time of admission? (open answer)**  None  <5  5-10  >10  Missing | 4 (13.8)  9 (31.1)  4 (13.8)  7 (24.1)  5 (17.2) | 1 (11.1)  3 (33.3)  0  4 (44.4)  1 (11.1) | 2 (5.0)  11 (27.5)  8 (20.0)  17 (42.5)  2 (5.0) | NS | NS | NS | NS |
| **How many children with bronchiolitis were older than 28 days of life or 44 weeks postconceptional age at the time of admission? (open answer)**  None  <5  5-10  >10  missing | 4 (13.8)  5 (17.2)  6 (20.7)  8 (27.6)  6 (20.7) | 2 (22.2)  4 (44.4)  1 (11.1)  1 (11.1)  1 (11.1) | 6 (15.0)  14 (35.0)  9 (22.5)  10 (25.0)  1 (2.5) | NS | NS | NS | NS |
| **In how many of these children with bronchiolitis was RSV isolated?**  None  1-10  11-20  21-30  >30  missing | 3 (10.3)  12 (41.4)  4 (13.8)  4 (13.8)  1 (3.4)  5 (17.2) | 1 (11.1)  4 (44.4)  2 (22.2)  0  1 (11.1)  1 (11.1) | 4 (10.0)  15 (37.5)  15 (37.5)  3 (7.5)  2 (5.0)  1 (2.5) | NS | NS | NS | NS |
| **What was the period of highest incidence of hospitalizations for RSV-positive bronchiolitis?**  January-February  March-May  October-December  Missing | 2 (6.9)  0  22 (75.8)  5 (17.2) | 0  0  7 (77.8)  2 (22.2) | 8 (20.0)  2 (5.0)  28 (70.0)  2 (5.0) | NS | NS | NS | NS |
| **To which of the following categories do the RSV+ patients admitted to your unit belong?**  Born in your hospital (including ex-premature infants)  Born in other hospitals and later transferred (including ex-premature infants)  Admitted from the Emergency Department/Pediatrics of your hospital  Transferred from the Emergency Department/Pediatrics of another hospital | 1 (2.2)  13 (28.9)  20 (44.4)  11 (24.4) | 5 (25.0)  4 (20.0)  7 (35.0)  4 (20.0) | 6 (8.9)  18 (26.8)  22 (32.8)  21 (31.3) | NS | NS | **0.04*** | NS |
| **What was the reason for ICU admission of children with RSV+ bronchiolitis?**  Hypoxia  Moderate-to-severe respiratory distress requiring ventilatory support (CPAP or invasive ventilation)  Apnea episodes  Feeding difficulties and dehydration  Risk factors (e.g., bronchopulmonary dysplasia, congenital heart disease, neuropathy, prematurity) | 13 (19.4)  23 (34.3)  16 (23.8)  10 (14.9)  5 (7.4) | 5 (23.8)  6 (28.5)  3 (14.2)  5 (23.8)  2 (9.5) | 13 (13.9)  34 (36.5)  14 (15.0)  25 (26.8)  7 (7.5) | NS | NS | NS | NS |
| **What type of ventilatory support did you predominantly use to treat RSV+ bronchiolitis patients?**  Low-flow oxygen therapy  High-flow oxygen therapy with nasal cannulas (HFNC)  Non-invasive ventilation  Invasive ventilation | 2 (4.2)  22 (46.8)  18 (38.3)  5 (10.6) | 5 (33.3)  6 (40.0)  4 (26.6)  0 | 9 (14.5)  32 (51.6)  19 (30.6)  2 (3.2) | **0.05*** | NS | **0.01**** | NS |
| **How many children were treated with non-invasive ventilatory support other than High Flow Nasal Cannula (HFNC)? (open answer)**  None  <5  5-10  >10  missing | 2 (6.9)  7 (24.1)  8 (27.6)  3 (10.3)  9 (31.0) | 2 (22.2)  5 (55.6)  0  0  2 (22.2) | 7 (17.5)  14 (35.0)  8 (20.0)  10 (25.0)  1 (2.5) | NS | **0.01**** | NS | NS |
| **How many children were treated with invasive ventilatory support? (open answer)**  None  <5  5-10  >10  missing | 10 (76.9)  2 (15.4)  0  0  1 (7.7) | 18 (41.9)  16 (37.2)  1 (2.3)  1 (2.3)  7 (16.2) | 11 (50.0)  8 (36.4)  0  0  3 (13.6) | NS | NS | NS | NS |
| **What pharmacological therapies did you use to treat RSV+ bronchiolitis patients?**  No pharmacological therapy, only ventilatory support  Hypertonic saline solution  Systemic steroids  Inhaled steroids  Inhaled bronchodilators  Endotracheal surfactant  Antibiotic therapy  Inotropes | 8 (13.8)  9 (15.5)  9 (15.5)  10 (17.2)  9 (15.5)  1 (1.7)  11 (18.9)  1 (1.7) | 4 (20.0)  4 (20.0)  3 (15.0)  2 (10.0)  3 (15.0)  0  4 (20.0)  0 | 14 (15.4)  24 (26.4)  9 (9.9)  19 (20.8)  10 (10.9)  2 (2.2)  12 (13.1)  1 (1.0) | NS | NS | NS | NS |
| **Did you use sedation for RSV+ bronchiolitis patients on non-invasive ventilatory support? If yes, which one?**  None  Midazolam  Dexmedetomidina  missing | 17 (58.6)  1 (3.4)  4 (13.8)  7 (24.1) | 7 (77.8)  0  0  2 (22.2) | 37 (92.5)  2 (5.0)  0  1 (2.5) | **0.05*** | NS | **0.03*** | NS |
| **What was the average length of stay (in days) in the NICU for children with RSV+ bronchiolitis? (open answer)**  None  <5  5-10  >10  Missing | 1 (3.4)  7 (24.1)  11 (37.9)  0  10 (34.5) | 0  3 (33.3)  4 (44.4)  0  2 (22.2) | 1 (2.5)  14 (34.0)  22 (55.0)  2 (5.0)  1 (2.5) | NS | NS | NS | NS |
